# Expression patterns of *Neil3 *during embryonic brain development and neoplasia

**DOI:** 10.1186/1471-2202-10-45

**Published:** 2009-05-09

**Authors:** Gunn A Hildrestrand, Christine G Neurauter, Dzung B Diep, Cesilie G Castellanos, Stefan Krauss, Magnar Bjørås, Luisa Luna

**Affiliations:** 1 Centre for Molecular Biology and Neuroscience, Department of Molecular Biology, Institute of Medical Microbiology, Rikshospitalet, Oslo University Hospital, Oslo, Norway; 2 Department of Chemistry, Biotechnology and Food Science, Norwegian University of Life Sciences, Ås, Norway; 3 The Norwegian School of Veterinary Science, Department of Production Animal Clinical Science, Oslo, Norway

## Abstract

**Background:**

The base excision repair pathway is responsible for repairing small DNA base lesions caused by endogenous and exogenous damaging agents. Repair is initiated by DNA glycosylases that recognize and remove the lesions. NEIL3 is one of 11 mammalian DNA glycosylases identified to date and it was discovered on the basis of sequence homology to the *E. coli *Fpg and Nei glycosylases. Difficulties in purifying the protein have limited its biochemical characterization and in contrast to the other glycosylases, its function remains unclear.

**Results:**

In this study we describe the expression pattern of *Neil3 *during mouse embryonic development with special focus on brain development. We have also looked at the expression of *NEIL3 *in several normal and tumor tissues. Quantitative real-time PCR and *in situ *hybridization revealed that *Neil3 *was highly expressed at embryonic days 12–13, when neurogenesis starts. The expression decreased during development and in the adult brain,*Neil3 *could not be detected in any of the brain areas examined by quantitative real-time PCR. During embryogenesis and in newborn mice specific expression was observed in areas known to harbour neural stem and progenitor cells such as the subventricular zone and the dentate gyrus. Finally, *NEIL3 *expression was higher in tumors compared to normal tissues, except for testis and pancreas.

**Conclusion:**

Our findings indicate that mammalian NEIL3 is specifically expressed in brain areas where neurogenesis takes place during development and that its expression is tightly regulated both temporally and spatially. In addition, *NEIL3 *seems to be upregulated in tumor tissues compared to normal tissues. Altogether, mammalian *NEIL3 *seems to be highly expressed in cells with high proliferative potential.

## Background

The base excision repair (BER) pathway, one of the major DNA repair pathways, is dedicated to the repair of damaged DNA bases arising from endogenous and exogenous insults [1-4]. The DNA bases may be subjected to oxidation, alkylation and deamination and oxidative DNA damage, in particular, has been implicated in the pathogenesis of many diseases including cancer, atherosclerosis, neurodegenerative diseases such as Parkinson's and Alzheimer's, and even aging [5-8]. DNA glycosylases are the key enzymes of BER; they initiate repair by catalysing the hydrolysis of the N-glycosylic bond between modified bases and the sugar-phosphate backbone [1,3,4,9]. To date, four mammalian DNA glycosylases that recognize and excise oxidized bases have been thoroughly characterized: OGG1, NTH1, NEIL1 and NEIL2 [10-12]. NEIL3 was identified together with NEIL1 and NEIL2 as a gene product with significant structural similarities to the *E. coli *Fpg and Nei DNA glycosylases [13-15]. However, in contrast to NEIL1 and NEIL2 no substantial DNA glycosylase activity has been detected [13,14,16,17]. Expression of human *NEIL3 *has been reported in thymus and testis, while mouse *Neil3 *has been demonstrated to be highly expressed in spleen, bone marrow, thymus, blood cells and brain regions that harbour progenitor cells [13,18,19]. *NEIL3 *has also been shown to be highly expressed in primary malignant melanomas associated with metastasis [20]. Most studies have been performed on adult organs and a recent paper described *Neil3 *expression in the developing mouse [17]. Here, we describe the expression pattern of *Neil3 *during embryonic development in mice with focus on the expression of *Neil3 *during brain development. The expression of *NEIL3 *in multiple human cancers was also examined.

## Results

### Neil3 is expressed during embryogenesis

**Figure 1:**
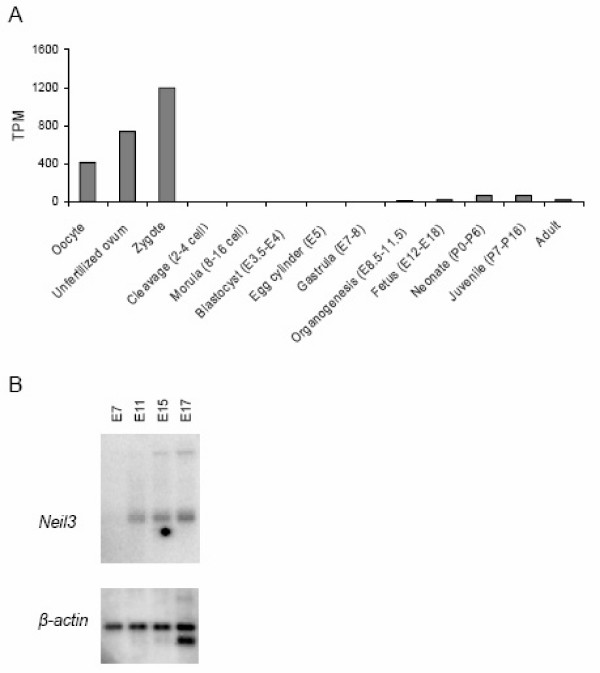
**Expression analysis of *Neil3 *during embryonic development**. (A) Expression profiles of *Neil3 *based on the EST profile viewer at the NCBI homepage. TPM, transcripts per million. (B) A multiple tissue northern blot containing poly(A)^+ ^RNA from mouse embryo tissues at four different developmental stages was hybridized with ^32^P-labeled cDNA probes for *Neil3 *and *β-actin*.

The temporal expression pattern of *Neil3 *during embryonic development was examined by *in silico *electronic Northern blot (eNorthern) and Northern blot analysis. We used the UniGene collection  to generate *in silico *expression profiles for *Neil3 *at different stages during embryonic development. As seen in Figure 1A, there is a very high expression in the oocyte, unfertilised oocyte and zygote and this expression dramatically falls after the zygote stadium. During embryogenesis, *Neil3 *transcription is not detected until E8.5–11.5. Northern blot analysis was performed by using a northern blot containing poly(A)^+ ^RNA isolated from whole mouse embryos at days 7, 11, 15 and 17 of gestation. Expression of *Neil3 *was not observed until E11 (Figure 1B).

### Neil3 is highly expressed in developing brain

**Figure 2:**
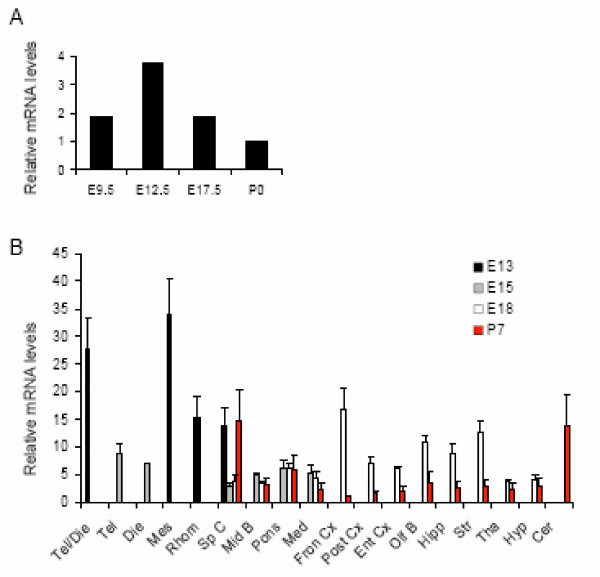
**Expression analysis of *Neil3 *in embryonic brain development**. (A) Real-time quantitative PCR was performed on mRNA isolated from mouse brains at four different developmental stages. The data were normalized to *Gapdh *and expressed relative to the level of *Neil3 *at P0. (B) A detailed expression profile of *Neil3 *during brain development was obtained by using a mouse developmental tissue qPCR array. The array contains one concentration of cDNAs, normalized to Gapdh. *Neil3 *mRNA levels were calculated relative to the sample expressing the lowest level of *Neil3 *(Fron Cx, P7). The experiment was conducted twice and the standard deviations are indicated. Abbreviations: Tel, telencephalon; Die, diencephalon; Mes, mesencephalon (midbrain); Rhom, Rhombencephalon; Sp C, spinal cord; Mid B, midbrain; Med, medulla; Fron Cx, frontal cortex; Post Cx, posterior cortex; Ent Cx, entorhinal cortex; Olf B, olfactory bulb; Hipp, hippocampus; Str, Striatum; Tha, thalamus; Hyp, hypothalamus; Cer, cerebellum.

Next, we examined the expression of *Neil3 *in developing mouse brain. Messenger RNA was isolated from brains at days 9.5, 12.5 and 17.5 of gestation and at P0 (birth) and subjected to quantitative real-time PCR. Figure 2A shows the expression levels of *Neil3 *relative to *Gapdh*. The highest expression level of *Neil3 *was observed at E12.5. To dissect the sites of *Neil3 *expression, we evaluated the distribution of *Neil3 *by transcript profiling in various brain regions at five different stages of development: E13, E15 and E18 (embryo), P7 (neonatal) and 5 weeks (adult). As seen in Figure 2B, *Neil3 *expression was highest at E13 in the fore- and midbrain and the expression decreased during development. In neonatal mice the expression was relatively low in all areas examined, except for in the cerebellum and the spinal cord. In adult mice, *Neil3 *expression was below the detection limit (data not shown).

### Neil 3 is highly expressed in regions of active neurogenesis in the developing brain

**Figure 3:**
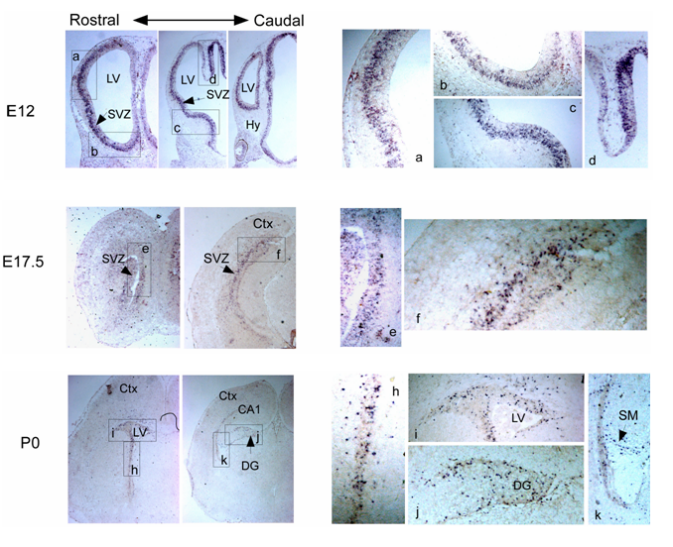
**Expression of *Neil3 *in developing mouse forebrain**. *In situ *hybridisation was performed on coronal sections of mouse brains at different embryonic stages. Magnifications of *Neil3*-positive cells are shown to the right. Abbreviations: CA, hippocampal differentiation fields; Ctx, cortex; DG, dentate gyrus; Hy, hypothalamus; LV, lateral ventricle; SM, secondary matrix; SVZ, subventricle zone.

In order to characterize in more detail the expression of *Neil3 *mRNA in developing brain, *in situ *hybridization was performed on foetal mouse forebrains at E12, E17.5 and P0 (Figure 3). At E12 abundant *Neil3 *mRNA was detected in the subventricular zone (SVZ) of the lateral ventricles (LV). The expression of *Neil3 *decreased during development and at E17.5 and P0, expression was limited to distinct cells in the cortical SVZ, in cells of the secondary matrix (SM), the dentate gyrus migratory route (DGM) and the dentate gyrus (DG). Thus, *Neil3 *was expressed in regions that are rich in progenitor cells. No signals were detected for the sense DIG-RNA probe used as a control (data no shown).

### NEIL3 is expressed in various tumor samples

**Figure 4:**
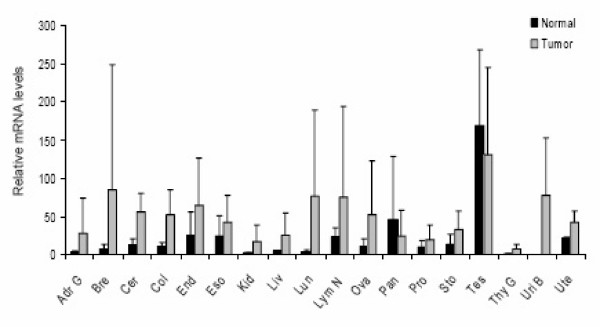
**Expression of *NEIL3 *in human cancers**. Transcript profiling was performed by using a disease tissue qPCR array. cDNA concentrations are normalized to β-actin. *Neil3 *mRNA levels are calculated relative to the sample expressing the lowest level of *Neil3 *(one of the stomach cancer samples). Tumor and normal tissue samples were grouped according to their origin and the average and standard deviations were calculated for each group. The experiment was conducted twice and the results from one of the experiments are shown. Abbreviations: Adr G, adrenal gland; Bre, breast; Cer, cervix; Col, colon; End, endometrium; Eso, esophagus, Kid, kidney; Liv, liver; Lun, lung; Lym N, lymph node; Ova, ovary; Pan, pancreas; Pro, prostate; Sto, stomach; Tes, testis; Thy G, thyroid gland; Uri B, urinary bladder; Ute, uterus.

*NEIL3 *has recently been shown to be highly expressed in melanomas [20]. We therefore investigated the expression of *NEIL3 *in several tumor types and their normal counterparts by using qPCR arrays containing cDNA from diseased and normal tissues. Data shown in Figure 4 is from one of the two experiments performed and represents relative *NEIL3 *mRNA levels in normal and tumor tissues. Raw data and information about the samples examined are shown in Additional file 1. Results revealed that although we observed high variation within the different cancer samples from the same tissue, tumor samples in general displayed a higher expression of *NEIL3 *than the corresponding normal tissues, except for testis and pancreas. Furthermore, *NEIL3 *was highly abundant in normal testis tissue, as has been described before [13].

## Discussion

In this study the expression of *Neil3 *during embryonic brain development was investigated. Our results showed that *Neil3 *was not uniformly expressed in the brain, but limited to specific cells in certain regions. The expression appeared to be tightly regulated both spatially and temporally during brain development.

Results from Northern blot analysis of total embryos revealed the presence of *Neil3 *after E7, while RT-PCR experiments showed the presence of *Neil3 *in the developing brain from E9. Thus, the expression of *Neil3 *appears to coincide with organogenesis. This is also supported by the *in silico *analysis, where *Neil3 *transcripts were not detected until E8.5–E11.5. Intriguingly, the *in silico *analysis revealed high expression of *Neil3 *during preimplantation. Mammalian embryos undergo major changes in their gene expression patterns throughout most stages of preimplantation development and massive maternal degradation of mRNA characterizes the transition from oocyte to early embryo before the onset of new transcription [21,22]. Genes that follow this expression pattern are suggested to have specific functions either in oogenesis, oocyte maturation, fertilization, and/or early phases of preimplantation development [23-25]. Our results suggest that *Neil3 *can be classified with this group of genes since *Neil3 *mRNA is highly expressed in unfertilised eggs and is abundant up to the zygote stadium before it is apparently degraded.

In mice, embryonic neurogenesis occurs from E12 to E17 [26,27]. There are two major proliferative populations in the developing brain [28]. The first to appear is a region called the ventricular zone (VZ) in the lateral ventricle. The VZ is lined by a population of cells that contains multipotent neural stem cells that give rise to most neurons and glial cells of the cortex. These cortical precursor cells constitute a heterogeneous population and there is increasing evidence that different precursor cells generate distinct differentiated cell types [29-33]. Another proliferative population is found in the subventricular zone (SVZ), located adjacent to the VZ along the lateral ventricle. Notably, neurogenesis continues in this region throughout adulthood, and in the adult mammalian brain, new neurons born in the SVZ migrate anteriorly into the olfactory bulb (OB), where they mature [34-36]. In post-natal mice, a second germinal zone exists; the subgranular zone (SGZ) of dentate gyrus in the hippocampus [36,37]. Our *in situ *results showed that during brain development *Neil3 *was highly expressed in cells found in places rich in neural progenitors such as the VZ and the SVZ. The expression decreased during embryonic development and at P0, only a few cells around the lateral ventricles and in the dentate gyrus of the hippocampus were positive for *Neil3 *expression. We have recently described the distribution of *Neil3 *during postnatal development and found *Neil3 *expression in the SVZ, the rostral migratory stream (RMS), the dentate gyrus of the hippocampal formation and the Purkinje cells of the cerebellum in P3 mice. The expression of *Neil3 *decreased dramatically in postnatal mice so that in 1-month-old mice only a few cells in the SVZ and in layer V of neocortex were detected. In 1-year-old mice *Neil3 *was detected in layer V of neocortex only [19]. In the present study no *Neil3 *expression was detected in brains from 5-week-old mice when using quantitative real-time PCR. The discrepancy between our results and the previously reported data could be due to a lower sensitivity of real-time PCR compared to *in situ *hybridization. Thus, the limited number of *Neil3*-positive cells detected in 1-month-old mice by *in situ *hybridization is probably below the detection limit of the real-time PCR. Altogether, previous data and results presented here suggest that *Neil3 *is highly expressed in regions where neurogenesis occurs during embryogenesis and to a lesser extent in neonatal animals [19]. Hippocampal *Neil3 *expression disappeared in 1-month-old mice but could still be observed in the SVZ [19]. This may suggest the existence of different progenitors in the two germinal areas. Since *Neil3 *appears to be enhanced in proliferating cells, the decline in *Neil3 *expression during development could be due to a decline in cell proliferation.

*Neil3 *knockout mice were generated a few years ago and these mice are viable and remain apparently healthy into adulthood with no overt phenotype [18]. A putative role in lymphocytes and/or other immune cells has been proposed since *Neil3 *is highly expressed in lymphatic cells and tissues and the knockout mice have a slightly reduced number of blood cells [18]. The expression pattern described in this paper warrants a closer examination of the distribution/phenotype of neural progenitor cells in the embryo and adult *Neil3 *knockout mice. Studies using the *Neil3 *knockout mice model that involve insults to the brain such as ischemic stroke would be central in revealing whether Neil3 has a role in the regeneration of damaged tissue.

In a recent study, *NEIL3 *was shown to be overexpressed in primary melanomas giving rise to metastasis [20]. *NEIL3 *was therefore suggested to be associated with the progression of primary melanoma to distant metastasis [20]. Interestingly, we found increased *NEIL3 *expression in 16 of 18 cancer tissues compared to normal tissues. Intriguingly, *NEIL3 *has also recently been suggested to be a host factor for HIV and thus, a target for antiviral medicines [38]. Taken together all these observations and the fact that no robust DNA glycosylase activity has been detected [16,17], indicate that NEIL3 is not a typical DNA glycosylase but rather a protein involved in processes where extensive proliferation is a hallmark such as brain development and possibly tumor development/progression.

## Conclusion

We have characterized the expression pattern of *Neil 3 *during embryonic development. Our results clearly showed that the expression of *Neil3 *appeared to be tightly regulated both temporally and spatially. High expression was detected in the oocytes and preimplantation stages of development and later during organogenesis. In the embryonic brain, *Neil3 *was detected in sites rich in neural stem/progenitor cells. Finally, tumor tissues were shown to express higher amounts of NEIL3 compared to their normal counterparts, except for testis and pancreas. These results call for a closer examination of the brains of *Neil3 *knock-out mice not only during development and aging, but also under detrimental conditions such as ischemic stress.

## Methods

### Northern Blot Analysis

A mouse embryo MTN blot purchased from Clontech (catalog no. 7763-1) was probed for *Neil3 *expression. Northern blot hybridization was carried out using ExpressHyb solution (Clontech) and probes were labeled using the Rediprime DNA labeling system (Amersham Biosciences), both according to the manufacturer's protocol. The full-length murine cDNA was used as a probe.

### Real-time Quantitative PCR

Messenger RNA was isolated from the brains of embryos and newborn mice (mixed background: 50% 129Sv and 50% C57BL/6) using the Dynabeads mRNA DIRECT Kit (Dynal). The RNA was treated with TurboDNase (Applied Biosystems) and cDNA synthesized using the High-Capacity cDNA Reverse Transcription kit (Applied Biosytems). The expression of glyceraldehyde-3-phosphate dehydrogenase (*Gapdh*) mRNA was used as endogenous control. Primers used: mouse Gapdh, forward 5'-TCG TCC CGT AGA CAA AAT GGT-3'-, reverse 5'-CGC CCA ATA CGG CCA AA-3'. Mouse Neil3, forward 5'-GGG CAA CAT CAT CAA AAA TGA A-3', reverse 5'-CTG TTT GTC TGA TAG TTG ACA CAC CTT-3'. Quantitative real-time PCR was performed in 20-μl reactions containing 20 ng of cDNA using the Power SYBR Green PCR master mix and the Step One Plus real-time PCR system (both from Applied Biosystems) according to the system and kit instructions. Relative gene expression was calculated using the comparative CT method and primers were designed using the Primer Express software version 2.0 (Applied Biosystems).

To investigate more specifically the expression of *Neil3 *in the developing mouse brain, a Mouse Developmental Tissue qPCR array (MDRT101; OriGene Technologies) was used. *Neil3 *primers used were the same as mentioned above. The array contained normalized cDNA prepared from various brain regions at five developmental stages. The cDNAs were normalized against *Gapdh*. Relative gene expression was calculated using the comparative CT method. The data presented are relative *Neil3 *mRNA levels.

For measurement of *NEIL3 *mRNA expression in normal and tumor tissues, we used a Disease Tissue qPCR array (CSRT303; OriGene Technologies). This array consisted of normalized cDNA prepared from pathologist-verified human tumor tissues obtained from 18 different tissues. The cDNAs were normalized against *β-actin*. Clinical information associated with each of these samples can be found in Additional file 1. Primers used: Human NEIL3, forward 5'-GGT CTC CAC CCA GCT GTT AAA G-3', reverse 5'-CAC GTA TCA TTT TCA TGA GGT GAT G-3'. Two parallel experiments were run and the raw data is presented in the additional file 1. Relative gene expression was calculated using the comparative CT method. The data presented are relative *NEIL3 *mRNA levels.

### In situ hybridization

For characterization of *Neil3 *gene expression in the brain, wild type (C57BL6/CBA F1 strain) embryos and newborn mice were used. Coronal cryosections were prepared and *in situ *hybridization was performed as previously described [39]. A plasmid containing full-length mNeil3 (clone 4945750; Invitrogen,) was linearized with appropriate enzymes before sense and antisense riboprobes were synthesized using a DIG RNA labeling kit (SP6/T7) (catalog no. 1175025; Roche).

## Authors' contributions

GAH, performed Northern Blot analysis, took care of the mice and removed brains from mice for RNA isolation, isolated RNA, discussed the results and contributed to the editing of the manuscript. CGN, performed real-time RT-PCR. DBD, performed *in situ *hybridization. CGC, took care of the mice and helped removed brains from mice for RNA isolation. SK, contributed to the planning of the experiment and provided financial support. MB, contributed to the planning of the experiment, discussed results, contributed to the editing of the manuscript and provided financial support. LL, conceived and supervised the experiments, performed the electronic Northern blot, discussed results and prepared the manuscript.

## Supplementary Material

Additional file 1 **Raw data of *NEIL3 *mRNA expression in normal and tumor tissues**. The data provided are the measurement of *NEIL3 *mRNA expression in normal and tumor tissues, we used a Disease Tissue qPCR array (CSRT303; OriGene Technologies). This array consisted of normalized cDNA prepared from pathologist-verified human tumor tissues obtained from 18 different tissues. The cDNA concentrations were normalized to β-actin. Raw data from two parallel experiments and information about the samples examined are shown in this file. Click here for file
